# Early periprosthetic hip joint infection managed by cementless one-stage revision – a case series

**DOI:** 10.5194/jbji-7-43-2022

**Published:** 2022-02-25

**Authors:** Kristoffer Riemer, Jeppe Lange

**Affiliations:** 1 Elective Surgery Center, Silkeborg Regional Hospital, HE Midt, Silkeborg, 8600, Denmark; 2 Department of Orthopaedic Surgery, Horsens Regional Hospital, Horsens, 8700, Denmark

## Abstract

**Background**: Early periprosthetic hip joint infection (PJI) is traditionally treated with debridement, antibiotics, and implant retention (DAIR). However, infection control rates after DAIR-treated periprosthetic hip joint infection do not exceed 77 %. Cementless one-stage revision of chronic PJI by the Cementless One-stage Revision of Infected Hip Arthroplasty (CORIHA) protocol has been evaluated positively with a 91 % success rate. We wanted to evaluate the effectiveness of cementless one-stage revision following the CORIHA protocol for early PJI in elective primary total hip arthroplasty, regarding risk of re-operation with exchange of implants. **Methods**: We identified 18 patients in our center with early ( ≤6 -week postoperative) PJI after primary total hip arthroplasty (THA) treated with one-stage cementless revision in the period January 2012–March 2018. Treatment followed the CORIHA protocol. Primary outcome was retention of implants at the most recent follow-up. Patients were followed for a minimum of 3 years. **Results**: Mean follow-up time was 60 months (39–105). All patients retained their implants, but two required superficial soft tissue debridement due to persistent wound seepage. **Conclusion**: Cementless one-stage revision appears to be an effective treatment of early PJI after primary THA and at least an equal choice of treatment compared with DAIR. Whether the potential benefit of a lower re-revision rate for postoperative PJI outweighs the increased surgical complexity of the CORIHA procedure needs further evaluation.

## 1 Introduction

Early​​​​​​​ (acute postoperative) periprosthetic hip joint infection (PJI) is traditionally treated with debridement, antibiotics, and implant retention (DAIR). However, infection control rates after DAIR-treated periprosthetic hip joint infection do not exceed 77 % (Tsang et al., 2017; Kunutsor et al., 2018).

Even repeated DAIR does not necessarily improve these results and furthermore requires an extra operation (Moojen et al., 2014; Chung et al., 2019).

A current alternative to the DAIR procedure in early PJI is exchange revision. Being able to control the infection in early PJI with one surgical procedure is optimal. So far there is limited knowledge about the effectiveness of one-stage revision in early PJI in the hip. In a paper from 2013 Hansen showed 70 % infection control with cementless one-stage revision for early PJI (Hansen et al., 2013). Since the publication of the Hansen paper knowledge of the importance of biofilm eradication in the surgical revision procedure has evolved immensely. Fully mature biofilm may form within 3–5 d. This must be taken into consideration when performing the surgical revision procedure.

As a result of the growing body of evidence, one-stage revision in late (chronic) PJI is gaining ground worldwide. Cementless one-stage revision following the Cementless One-stage Revision of Infected Hip Arthroplasty (CORIHA) protocol has shown promising results in chronic PJI with 91 % infection control, even in cases with fistulation and/or preoperative unknown microorganisms (Lange et al., 2018). In this protocol there is a special focus on appropriate soft tissue and bone debridement to ensure surgical biofilm eradication with an appropriate anti-biofilm postoperative antibiotic regimen based on antibiograms. Based on the preliminary results obtained in the CORIHA protocol in chronic PJI, the CORIHA protocol has been applied in our high-volume elective surgery center since 2012 and is now the first-line procedure for all PJI, early or late.

**Figure 1:**
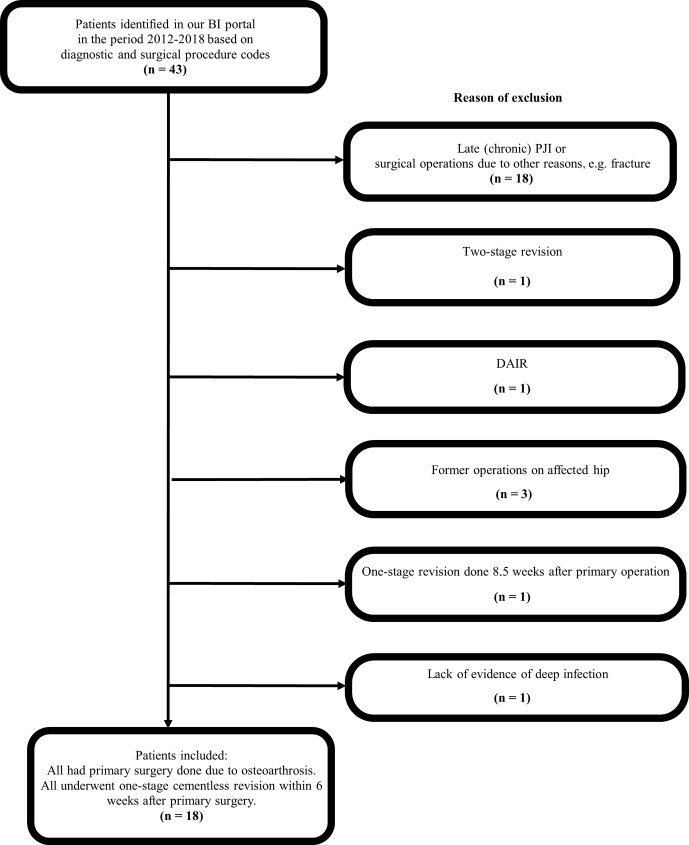
Flow diagram of patient selection.

The purpose of this study is to evaluate the effectiveness of cementless one-stage revision following the CORIHA protocol for early PJI in elective primary total hip arthroplasty (THA) regarding risk of re-operation with exchange of implants.

## 2 Methods

### 2.1 Study design

This is a retrospective case series.

### 2.2 Study setting

All healthcare services are free, with equal and unrestricted access, due to the national 100 % publicly funded healthcare system. Our center has a tertiary referral function in revision THA and is one of six public orthopedic surgical centers in our region. The local region covers a catchment area of approximately 1.2 million inhabitants on which data are captured. To ensure a minimum of 3 years of follow-up, patients were identified from the period between January 2012 and March 2018. Approximately 5000 elective THA and 500 aseptic and septic revisions were performed at our center in the study period, with cementless implants used in the vast majority (Gundtoft et al., 2016) (https://danskhoftealloplastikregister.dk/en/dhr/, last access: 5 July 2021).

All revisions in the study period were performed by three senior consultant hip joint replacement surgeons. All revisions following the CORIHA protocol were performed by a single surgeon.

## 3 Patients

We identified patients via our regional business intelligence portal, where all regional patient contacts, including diagnosis and surgical procedures, are registered on a day-to-day basis. Diagnosis is based on the WHO's International Statistical Classification of Disease and Related Health Problems 10th Revision and Procedure based on the Nordic NCSP NOMESCO Classification of Surgical Procedures. The following codes were used for identification: diagnosis code DT845 (infection and inflammatory reaction due to internal joint prosthesis) in combination with procedure codes KNFC20 (secondary insertion of both components of uncemented total prosthesis in hip joint) and KNFW69 (re-operation in case of deep infection after surgery on hip or thigh). The surgeon performing the revision procedures following the CORIHA protocol used a uniform coding strategy in the study period, and as such all relevant patients are believed to be identified within this search.

Patients were included in our case series (see Fig. 1) if the index procedure was an elective primary cementless THA performed due to osteoarthrosis with no previous registered surgery to the hip, and they were revised for an early PJI in relation to this index procedure.

In this study early PJI is defined as having the revision procedure done within 6 weeks of the index THA.

The preoperative definition of infection leading to revision surgery was at the surgeons' discretion in a case-by-case evaluation. All patients revised had raised C-reactive protein and leukocytosis. Clinical signs leading to the decision of revision were fever, wound seepage, reddening around the wound, and pain. Only one patient had fluid aspiration done prior to revision surgery.

For study purposes, PJI was defined as evidence of direct communication (fistulation) to the joint described during the revision procedure in case of negative cultures (3 patients) or ≥ two positive intraoperative tissue samples obtained from the hip joint (see below under procedure) with the same microorganism (15 patients) (McNally et al., 2021).

Electronic patient records, fully implemented on a regional basis since 2012, were retrospectively analyzed and data-extracted. The data extracted were baseline demographics at the time of revision and clinical characteristics including surgical procedure information (see Tables 1–3).

**Table 1:** Patient characteristics.

n	Age	Gender	BMI	Smoking	ASA	CCI	McPherson
	(years)		(kg m ${}^{-2}$ )	status		(score 0–29)	
1	63	F	39.7	No	2	2	IB1
2	52	M	42.9	FS	3	5	IB1
3	78	F	33.4	No	2	2	IB1
4	77	F	32.2	No	2	2	IB1
5	72	F	27.5	No	1	1	IA1
6	72	F	30.9	No	2	1	IA1
7	73	M	28.1	No	2	2	IA1
8	44	F	41.2	No	3	1	IA2
9	58	M	32.6	FS	2	3	IB1
10	76	M	29.3	No	2	1	IA2
11	82	M	27.2	FS	2	2	IB1
12	72	F	17.2	FS	2	1	IA1
13	78	F	21.5	FS	2	1	IA1
14	76	F	27.7	No	2	1	IA1
15	63	F	42.0	Yes	3	4	IC2
16	68	M	31.2	No	2	1	IA1
17	67	M	44.6	No	3	1	IA2
18	81	F	25.4	No	2	2	IB1

F: female, M: male, BMI: body mass index, FS: former smoker, ASA: Physical Status Classification System from the American Society of Anesthesiologists, CCI: Charlson comorbidity index.

The Charlson comorbidity index (CCI) was assessed with the use of an Internet-based calculator (https://orthotoolkit.com/charlson-comorbidity-index/, last access: 5 July 2021). Seventeen comorbidities associated with mortality are appropriately assigned, weights ranging from one to six points. The final score is obtained via the summation of applicable points and ranges from 0 (no disease burden) to 29 (maximal disease burden).

Patients were staged according to the McPherson staging system for prosthetic joint infection (McPherson et al., 2002).

All patients included were asked to report their Oxford hip score (OHS).

## 4 CORIHA protocol

All patients were treated with one-stage cementless revision according to the CORIHA protocol (Lange et al., 2018), with only minor differences.

The importance of adequate surgical debridement to clear any biofilm must be emphasized, as one cannot rely on the post-operative antibiotics to clear residual biofilm (Saeed et al., 2019).

Briefly, the CORIHA protocol requires the following.

- Excision of scar tissue.
- Acquisition of relevant tissue biopsies from the infectious punctum maximum near the bone–metal interface ad modum Kamme–Lindberg (Kamme and Lindberg, 1981​​​​​​​).
- Removal of all foreign matter, including well-fixed implants.
- Meticulous biofilm-oriented debridement, including reaming of the femoral medullar canal performed to the level of the femur condyle and relevant reaming of the acetabulum.
- Irrigation with 6 L of sterile saline water and after this with 1 L sterile saline water containing 2 g vancomycin and 240 mg gentamicin.
- Insertion of the cementless implant.
- Placement of collagen fleeces containing gentamicin. One fleece must be placed in the femoral canal securely below the implant.
- Meticulous closure in relevant layers to avoid cavitation. No drain or pain catheters must be used.

All procedures were performed according to the CORIHA protocol, with two consistent differences.

- Drapes were changed and re-disinfection performed between removal of the primary implant and insertion of the revision implant.
- Only two collagen fleeces were used: one fleece of Septocoll E 80 (Biomet) was placed in the femoral canal and one in the joint.

Priority is given to a standardized anti-biofilm antibiotic regimen based on Zimmerli et al. (2004). However, the individual regimen is instituted at the surgeon's discretion based on the individual antibiogram. As soon as tissue biopsies are obtained, systemic prophylactic antibiotics are initiated. This antibiotic regimen initially consists of intravenous vancomycin combined with either intravenous dicloxacillin or intravenous cefuroxime. When a definitive antibiogram is present, the treatment is changed accordingly. After a minimum of 10 d of intravenous treatment, oral treatment is initiated. The protocol defines an antibiotic treatment for a total duration of 12 weeks.

The duration of intravenous and oral antibiotics roughly followed the recommendations from the CORIHA protocol, but four patients did not receive intravenous vancomycin, and the duration of oral antibiotics varied from 3 to 16 weeks of treatment (mean 9 weeks).

## 5 Follow-up

After discharge patients were seen at least monthly while on antibiotic treatment. Treatment with antibiotics was stopped when a clinical evaluation of the patient, performed by the operating surgeon, showed no local signs of infection/inflammation and C-reactive protein was below 10 mg L ${}^{-1}$ .

In May 2021 a medical record review for vital status and further registered treatments based on the regional electronic patient records was performed by the first author – giving a minimum of 3 years of follow-up.

## 6 Outcome measures

We defined the primary outcome of infection control as retention of revision implants at the most recent follow-up. Secondary outcomes were survival (all-cause mortality) and revisions for other causes than PJI in the follow-up period.

Patient-related outcome measures consisted of a validated version of the OHS questionnaire completed by the patients in May 2019.

## 7 Data analysis

Normal distribution is checked by plotting the data via Q–Q plots. Binary data are reported as proportions, normal distributed data as means with a minimum to maximum range due to a limited number of cases, and categorical or non-normal distributed data as a median with a minimum to maximum range.

**Table 2:** Intraoperative microbiology, antibiotic therapy, OHS, follow-up, and outcome.

n	Intraoperative cultures	Intravenous antibiotics	Oral antibiotics	Oxford	Follow-up	Outcome
		(d)	(d)	hip score	(months)	
1	*Corynebacteria species*	Ce (7)	Im + Ri (42)	46​​​​​​​	105	Retention
2	*S. aureus*	Ce (14)	Ri (21), Di (63), Ci (84)	17	80	Retention
3	*MRSA*	Ce (3), Va (6)	Ri (35), Ci (28)	NC	84	Retention
4	*Mixed flora*	Ce (5), Va (10)	Ri (35), Ci (28)	28	78	Retention
5	*No growth*	Ce (5)	Ri (14), Di (7), Ci (7), He (7)	19	71	Retention
6	*S. aureus*	Ce (12), Di (4)	Ri (56), Ci (42)	44	69	Retention
7	*No growth*	Ce (14), Ge (14)	Im (42)	30	63	Retention
8	*MRSA*	Ce (3), Va (14)	Mo + Ri (70)	NC	54	Retention
9	*S. aureus*	Ce (1), Va (1), Di, (14)	Ci + Ri (56)	45	59	Retention
10	*S. aureus*	Va (1), Di (14)	Pr (70)	48	48	Retention
11	*S. aureus*	Ce (14), Va (2)	Ri + Ci (84)	16	49	Retention
12	*S. aureus*	Ce (1), Va (1), Di (12)	Di (63)	48	49	Retention
13	*No growth*	Ce (5), Va (5), Di (10)	Di (84)	24	49	Retention
14	*S. aureus*, *GAS*	Ce (14), Va (3)	Bi (49), Di (35)	22	48	Retention
15	*S. aureus*	Ce (1), Va (1), Di (14)	Di (112)	35	40	Retention
16	*S. aureus*	Ce (2), Va (2), Di (10)	Di (70)	22	39	Retention
17	*P. multocida, CoNS*	Ce (3), Va (14), Am (11)	Ci + Ri (70)	45	39	Retention
18	*S. aureus*	Ce (14), Va (2)	Da (70)	36	41	Retention

CoNS: *coagulase-negative staphylococci*, GAS: *Group A Streptococcus*, MRSA: *methicillin-resistant Staphylococcus aureus*, Am: ampicillin, Bi: amoxicillin/clavulanic acid, Ce: cefuroxime, Ci: ciprofloxacin, Da: clindamycin, Di: dicloxacillin, Ge: gentamicin, He: flucloxacillin, Im: amoxicillin, Mo: moxifloxacin, Pr: phenoxymethylpenicillin, Ri: rifampicin, Va: vancomycin. Oxford hip score – we are using the new scoring system running from 0 to 48, with 48 being the best outcome. NC: not completed.

**Table 3:** Implants and surgery time.

n	Primary implants	Revision implants	Revision surgery
			time (min)
1	Stryker Trident Cup, DePuy Synthes Corail Stem	Stryker Trident Cup, DePuy Synthes Corail Stem	97
2	DePuy Synthes Pinnacle Gription Cup and Corail Stem	DePuy Synthes Pinnacle Gription Cup and Corail Stem	79
3	Stryker Trident Cup, DePuy Synthes Corail Stem	DePuy Synthes Pinnacle Cup and Corail Revision Stem	104
4	DePuy Synthes Pinnacle Cup and Corail Stem	DePuy Synthes Pinnacle Cup and Corail Stem	89
5	Stryker Trident Cup, DePuy Synthes Corail Stem	DePuy Synthes Pinnacle Gription Cup and Corail Stem	92
6	Stryker Trident Cup, DePuy Synthes Corail Stem	Stryker Trident Cup, DePuy Synthes Corail Stem	76
7	DePuy Synthes Pinnacle Cup and Corail Stem	DePuy Synthes Pinnacle Cup and Corail Revision Stem	95
8	DePuy Synthes Pinnacle Gription Cup and Corail Stem	DePuy Synthes Pinnacle Gription Cup and Corail Stem	90
9	DePuy Synthes Pinnacle Cup and Corail Stem	DePuy Synthes Pinnacle Cup and Corail Stem	90
10	DePuy Synthes Pinnacle Cup, Zimmer CLSSpotorno Stem	DePuy Synthes Pinnacle Cup, Zimmer CLS Spotorno Stem	82
11	DePuy Synthes Pinnacle Cup and Corail Stem	DePuy Synthes Pinnacle Gription Cup and Corail Revision Stem	99
12	DePuy Synthes Pinnacle Cup, Zimmer CLSSpotorno Stem	DePuy Synthes Pinnacle Gription Cup and Corail Stem	105
13	DePuy Synthes Pinnacle Cup and Corail Stem	DePuy Synthes Pinnacle Cup and Corail Stem	113
14	DePuy Synthes Pinnacle Cup, Zimmer CLSSpotorno Stem	DePuy Synthes Pinnacle Cup, Zimmer CLS Spotorno Stem	75
15	DePuy Synthes Pinnacle Cup and Corail Stem	DePuy Synthes Pinnacle Cup and Corail Stem	77
16	DePuy Synthes Pinnacle Cup and Corail Stem	DePuy Synthes Pinnacle Cup and Corail Stem	89
17	DePuy Synthes Pinnacle Gription Cup,ZimmerBiomet Wagner Cone Stem	DePuy Synthes Pinnacle Gription Cup, ZimmerBiomet Wagner Cone Stem	90
18	DePuy Synthes Pinnacle Cup and Corail Stem	DePuy Synthes Pinnacle Cup and Corail Stem	88

## 8 Results

### Patients

The series included 11 women (61 %) and 7 men (39 %). At the time of revision arthroplasty, the mean age was 70 years (44–82). Mean body mass index was 32 kg m ${}^{-2}$ (17–45). Twelve were non-smokers (67 %), five former smokers (28 %), and one an active smoker (5 %).

- The median American Society of Anesthesiologists Physical Status Classification System score was 2 (1–3).
- The median CCI was two points (1–4).
- The mean follow-up time was 60 months (39–105).
- Mean duration of revision surgery was 91 min (75–113). In none of the cases was a trochanteric osteotomy necessary to remove the femoral stem since solid bone ingrowth had not yet occurred.
- *Staphylococcus aureus* was the most frequently cultured bacterium ( n=11 ; 61 %). The mean duration of intravenous antibiotic treatment was 12.5 d (7–17). The mean duration of oral treatment was 64 d (21–112). The mean OHS was 33 (range 16–48).

Individual information on infecting microorganism, postoperative antibiotics, OHS, follow-up time, components used in the index, and revision arthroplasty is shown in Tables 2 and 3.

## 9 Main results

All patients retained their revised implants from the CORIHA protocol revision procedure. No patients had a medical chart description of chronic fistulation, and no patients received antibiotic treatment at the time of final follow-up. All patients were alive at the time of the final follow-up.

One patient had two subsequent operations due to periprosthetic fractures with negative tissue biopsies at both operations. The first operation was 1 month after revision surgery and the second operation 2 years after revision surgery. The patient was treated with open reduction and internal fixation with no exchange of the revision implants and no clinical signs of persistent infection.

Four patients had minor complications with prolonged wound healing. Two of these had signs of superficial wound infection, and wound revision was performed. In both, the surgeon found an intact and viable iliotibial fascial layer with no signs of penetrating infection and only revised the skin and subcutaneous layers. One of the patients had negative tissue biopsies at both primary revision and wound revision. The negative tissue biopsies at primary revision were most likely due to 5 d of IV treatment with cefuroxime prior to the operation. The other patient had growth of *Staphylococcus aureus* at primary revision. In tissue cultures from the wound revision there was growth of a few *Staphylococcus epidermidis* interpreted as skin contamination and treated with relevant antibiotics according to antibiograms. The third patient was primarily infected with *Staphylococcus aureus*.

Three of the four patients healed without problems within 6 months, with no further signs of infection. The fourth patient healed within 15 months; the markedly prolonged wound healing was attributed to possible damage to a venous blood vessel and significant venous insufficiency combined with hypertension and atrial fibrillation (necessitating anticoagulant therapy), which resulted in severe edema of the lower extremities. The infecting microorganisms were *P. multocida* and *coagulase-negative Staphylococci*. None of the four cases has subsequently been clinically identified as suspect for chronic PJI.

## 10 Discussion

Based on our case series, we believe the CORIHA protocol describes an alternative to DAIR in early hip PJI in elective, cementless, and primary THA in patients with osteoarthrosis and no previous hip surgery.

All our patients were treated with success, yielding 100 % retention of revised implants and a clinically complete infection control.

Only a few studies exist on the topic of cementless, one-stage revision in early PJI of the hip joint. Hansen et al. (2013) achieved 70 % infection control in early PJI. Winkler et al. (2008) described 92 % infection control with cementless, one-stage revision using cancellous allograft bone impregnated with antibiotics. Twelve of 37 patients had early PJI, but separate results for this subgroup are not described. The use of cancellous bone graft makes the procedure described by Winkler et al. (2008) quite elaborate. Our protocol is easier to adapt and shows similar results. We believe that the collagen fleeces containing gentamicin can fully replace the bone graft.

In the paper by Wolf et al. (2014) a subgroup of 24 patients had cementless one-stage revision for early PJI. The infection control rate was 75 % (18 out of 24). They did not use local antibiotics intraoperatively but lavage with Betaisadona^®^.

Theoretically, removing all implants increases the likelihood of successful removal of biofilm. When implants are extracted, it is easier to perform soft tissue debridement of the anterior part of the joint and the femoral canal. The operation time is longer, and the surgical complexity increased in a one-stage revision when compared to DAIR, and we recommend that the procedure be only carried out by an experienced hip revision surgeon. However, it appears that there are no initial longevity concerns with the described complete exchange of implants. The low number of patients available for the study gives single-center, long-term follow-up some uncertainty, and international collaboration is warranted. We find it plausible that one-stage revision can supersede DAIR in early hip PJI in centers with relevant expertise regarding infection control.

According to the CORIHA protocol, antibiotic treatment should be continued for a minimum of 12 weeks. In nine of our patients the treatment period was shorter. However, no detrimental effect was detected, and it may be feasible to discontinue antibiotic treatment prior to 12 weeks as long as C-reactive protein has normalized and normal clinical conditions are present. Whether the change in drapes between removal of primary implants and insertion of revision implants contributes to the high level of infection control in our series is uncertain. This is relevant as the peri-operative change is logistically and economically demanding and needs to be further investigated.

Patient-related outcome measures are a very important parameter when claiming successful treatment. Six out of 16 patients completing the OHS questionnaire had satisfactory scores ≥39 (Galea et al., 2020). Three had intermediate results and seven unsatisfactory results. Two did not complete the questionnaire, one due to language problems and the other due to dementia. A mean OHS of 33 is similar to results of two other studies describing post-operative OHS after one-stage revision (Jenny et al., 2014; Kuiper et al., 2018).

This indicates that even though we have a 100 % success rate regarding the primary outcome with complete retention of implants, the treatment of PJI still has a major impact on these patients' lives regarding function and quality of life (Poulsen et al., 2017, 2019).

## 11 Limitations

Any retrospective case series is prone to both selection and information bias, and this needs to be taken into consideration when interpreting the results of our study. As such we cannot make conclusive remarks on the clinical effectiveness of the revision procedure described in the CORIHA protocol in early hip PJI. The number of patients in our case series is small, and just two patients with reinfection would have altered the results markedly. We believe we have found and included all eligible cases from our center but may have failed to identify relevant cases, although the coding praxis of the surgeon has not changed in the study period. Five patients with one-stage revision performed were excluded (see Fig. 1) from this case series, but it is noteworthy that all of them had infection control with retention of implants at follow-up (data not presented). Our study is a single-surgeon series, and the results obtained need to be confirmed by others. Cementless one-stage revision of early hip PJI following the CORIHA protocol could very easily be investigated with regard to reproducibility in other settings, and we would like to encourage the surgeons in the sub-specialty of hip surgery to do so.

## 12 Conclusion

Cementless one-stage revision appears to be a valid treatment of early PJI after elective primary THA in patients with osteoarthrosis and no previous surgery to the hip and at least an equal choice of treatment compared with DAIR. Whether the potential benefit of a potential lower re-revision rate outweighs the increased surgical complexity of the CORIHA procedure needs further evaluation.

## Data Availability

Raw data may be made available upon request to the corresponding author.
